# The Association between Type 2 Diabetes Mellitus and Women Cancer: The Epidemiological Evidences and Putative Mechanisms

**DOI:** 10.1155/2015/920618

**Published:** 2015-03-19

**Authors:** Kyong Hye Joung, Jae-Wook Jeong, Bon Jeong Ku

**Affiliations:** ^1^Department of Internal Medicine, Chungnam National University School of Medicine, 282 Munhwa-ro, Jung-gu, Daejeon 301-721, Republic of Korea; ^2^Department of Obstetrics, Gynecology and Reproductive Biology, Michigan State University, College of Human Medicine, 333 Bostwick Avenue NE, Grand Rapids, MI 49503, USA

## Abstract

Type 2 diabetes mellitus (T2DM), a chronic disease increasing rapidly worldwide, is well established as an important risk factor for various types of cancer. Although many factors impact the development of T2DM and cancer including sex, age, ethnicity, obesity, diet, physical activity levels, and environmental exposure, many epidemiological and experimental studies are gradually contributing to knowledge regarding the interrelationship between DM and cancer. The insulin resistance, hyperinsulinemia, and chronic inflammation associated with diabetes mellitus are all associated strongly with cancer. The changes in bioavailable ovarian steroid hormone that occur in diabetes mellitus (the increasing levels of estrogen and androgen and the decreasing level of progesterone) are also considered potentially carcinogenic conditions for the breast, endometrium, and ovaries in women. In addition, the interaction among insulin, insulin-like growth factors (IGFs), and ovarian steroid hormones, such as estrogen and progesterone, could act synergistically during cancer development. Here, we review the cancer-related mechanisms in T2DM, the epidemiological evidence linking T2DM and cancers in women, and the role of antidiabetic medication in these cancers.

## 1. Introduction

Diabetes mellitus (DM), mostly type 2 diabetes mellitus (T2DM), is one of the most common chronic diseases characterized by hyperglycemia. The World Health Organization (WHO) announced that the worldwide prevalence of DM in 2000 was 171 million and would reach approximately 366 million by 2030 [1]. However, the worldwide prevalence of DM has already reached 346 million as of 2010 [2]. The exponential growth and future burden of the high DM prevalence are responsible for most of the mortality and morbidity rates worldwide [3, 4]. Therefore, many studies have investigated the association and effects between DM and DM-related disease extensively, particularly the relationship between T2DM and cancer [5–8]. The recent consensus report sponsored by the American Association of Clinical Endocrinologists and the American College of Endocrinology (AACE/ACE Consensus Statement) highlighted that large and systemic studies are needed to investigate the relationship between T2DM and cancer [9].

The association between T2DM and cancer was reported more than 100 years ago [10]. Most epidemiological studies have suggested that cancers, particularly hepatic, pancreatic, colorectal, bladder, endometrial, and breast cancers, appear to be associated with T2DM, increasing the risk and mortality rates [1, 3, 6, 11–20]. Each value of the estimated risk may differ because of the impact of variable and intermingling factors, such as ethnic differences—including genetic susceptibility, life-style behavior, and environmental exposure—across populations [21]. However, recent studies have suggested that variable biological effects of diabetes may act synergistically with other definite cancer risk factors, particularly with ovarian steroid hormones [21–25]. Although the different impacts of ovarian steroid hormones and biological differences between males and females may lead to variations in cancer incidence, prognosis, and clinical outcomes, dedicated research on the effects of T2DM and cancer in females is limited.

In the present review, we will discuss the epidemiological evidence and possible mechanisms behind the relationship between T2DM and cancer in women, as well as the effect of diabetes treatments on cancer incidence and comorbidity.

## 2. Mechanisms of Carcinogenesis in Diabetes Mellitus

Carcinogenesis is a multistep process that undergoes various genetic “hits,” and diabetes may influence these processes by several mechanisms, particularly in females. DM and cancer share several common mechanisms, including increased insulin and insulin-like growth factor (IGF) signaling, dysregulation of ovarian steroid hormones, and chronic inflammation.

### 2.1. Insulin/IGF Signaling

Insulin and IGFs are well known for their involvement in cell survival and proliferation, as well as carbohydrate metabolism [26]. Unlike epidermal growth factor (EGF) and platelet-derived growth factor, which play roles at the cellular or tissue level as autocrine or paracrine factors, insulin and IGFs play important systemic regulatory roles at the whole organism level as a hormone [27–29]. Insulin and IGFs show hormonal effects through the insulin receptor (IR) and IGF receptors (IGFRs), which are widely expressed in normal tissues [30, 31]. Both types of receptors are membrane receptors with the tyrosine kinase domain located inside the cell membrane [32]. In terms of biological activity, these receptors form a holoreceptor characterized by two “half receptors,” which comprise an extracellular *α*-chain and an intracellular *β*-chain [32]. Half receptors of insulin exist as two splice variant isoforms, “A” and “B.” While the “B” [33] isoform recognizes only insulin, the “A” isoform recognizes both insulin and IGF-2 and is expressed most commonly by cancer cells [34]. The half IR and half IGF-1 receptor (IGF1R) can interact and form tetrameric structures known as “hybrid receptors,” which exert similar, but not identical, downstream signaling to that of IR or IGF1R [34–36]. Autoregulated or ligand-recognized IRs activate insulin receptor substrates (IRS) through tyrosine phosphorylation, thereby activating the phosphatidylinositol 3-kinase (PI3K) pathway and Ras/mitogen-activated protein kinase (MAPK) pathway known as mitogenic signaling by insulin [34].

Many epidemiological studies have suggested that insulin and IGF-1 play important roles in the regulation of cancer. An increased insulin or IGF-1 level, which presents in T2DM, obesity, and acromegaly, is strongly associated with increased cancer risk and mortality [37–41]. Some studies have shown evidence that the rates of insulin secretion among individuals may influence the risk and progression of cancer [42, 43]. Additionally, the insulin and IGF-1 levels in cancer patients are proportional to cancer-related mortality [26, 44]. Many in vitro and in vivo studies have shown evidence supporting epidemiological studies. For example, insulin or IGF-1 increased cell proliferation and reduced apoptosis in cancer cells, even at physiologically relevant concentrations [45, 46]. On the other hand, insulin signaling deficiency caused by the downregulation of IRs was shown to inhibit the proliferation and metastasis of cancer cells in vitro and in vivo [47, 48]. Insulin has direct access to its receptors, but most circulating IGFs are bound to IGF binding proteins (IGFBPs) and thereby demonstrate limited and attenuated IGFR-mediated bioactivity [49]. Therefore, the biological activity of IGFs may be determined by the level of IGFBPs, which are influenced by various conditions associated with insulin resistance such as T2DM and obesity [50–52]. Insulin resistance and increased insulin levels are associated with increased risk and mortality in women with cancer, particularly breast, endometrial, and ovarian cancers [53–55].

Although mainstream studies have suggested that insulin and IGFs are associated strongly with cancer development, each study showed a different degree of cancer risk associated with insulin and IGFs [33]. The latter view may be due to the following reasons. First, the functional differences in receptors may be due to the various types of tyrosine kinases, leading to phosphorylation of different IRS members. Therefore, insulin and IGFs may lead to various effects depending on the combination of half receptors in cancer cells. Second, the insulin and IGF cancer-related signaling pathways have adapted from those of normal cells, particularly from insulin-sensitive tissues such as the liver, muscle, and adipose tissue. However, the internal signals of a cancer cell are very different from those of normal cells because of the changes in genetic and/or epigenetic factors, thereby stimulating another signaling pathway in response to insulin and IGF. Therefore, insulin and IGFs may induce a cancer cell-specific signaling pathway aberrant from that of normal cells [26, 56].

### 2.2. Ovarian Steroid Hormone/Sex Hormone-Binding Globulin Regulation

The ovarian steroid hormones are some of the most common types of hormones related to cancer generation and/or progression. The predominating theories suggest that the ovarian steroid hormones estrogen and progesterone have a strong association with endometrial, breast, and ovarian cancers [57–59]. Enhanced signaling by estrogen in particular has been considered a risk factor for females with cancer. This is based on the observation that the estrogen increase and endometrial proliferation rate during the follicular phase of the menstrual cycle drive ductal elongation in mammary gland development during puberty [60–62].

Epidemiological studies have demonstrated that postmenopausal women are at an increased risk of cancers from exogenous estrogen replacement without progesterone [63–69]. Other studies have shown that polycystic ovarian syndrome (PCOS) in premenopausal women is very closely related to increased cancer risk and results from increased androgen and decreased progesterone levels [70–74]. The sex hormone-binding globulin (SHBG) level is one of the most important factors in cancer generation and/or development in postmenopausal women because its reduction leads to an increase in free ovarian steroid hormones [75–82].

Estrogen in cancer cells may trigger proliferation and cellular growth through the activation of estrogen receptor alpha (ER*α*) following the activation of PI3K and MAPK pathways [83, 84]. The role of estrogen is important because ovarian steroid hormone-sensitive tissues responding to ovarian steroid hormones exhibit increased levels of bioactive IGF-1 and gene expression of IGF1R, IRS-1, and IRS-2 [85–87]. In addition, activation of IR and IGF1R induces the phosphorylation of ER*α*, thereby potentiating ER*α* signaling [88, 89]. Therefore, cancer cells expressing higher levels of IR and/or IGF1R would result in resistance to antiestrogen therapy such as tamoxifen [89–91]. Although the role of androgen in promoting carcinogenesis and cell proliferation is well known in prostate cancer, in vitro studies have suggested that androgen could affect cell viability and proliferation through the regulation of inflammatory and Notch signaling pathways [92, 93].

Interestingly, hyperinsulinemia and/or insulin resistance, particularly in postmenopausal females with T2DM, result in increased bioavailable ovarian steroid hormone levels through suppressed hepatic SHBG production and induced ovarian steroid hormone production [23, 85, 94–97]. Additionally, the increased insulin and IGF-1 levels in females with T2DM potentiate ER*α* signaling by IR and/or IGF1R. Such observation and prediction suggest that diabetes, particularly T2DM, may involve cancer generation and/or development mechanisms through abnormal sex hormone signaling.

### 2.3. Chronic Inflammation

Most of our current knowledge indicates that the net effect of inflammation is the triggering of cancer development and progression [98–102]. Mediators of inflammatory pathways such as interleukin-6 (IL-6), tumor necrosis factor alpha (TNF*α*), and cyclooxygenase-2 (COX-2) are involved in cancer-related mechanisms that diminish tumor suppressor function, stimulate oncogene expression, and increase cell cycling [103]. Conversely, inhibition of inflammatory signaling such as that by nuclear factor kappa-light-chain-enhancer of activated B cell (NF-*κ*B) reduces cancer incidence [104, 105]. Additionally, many studies concerning the association of inflammation and cancer in females suggest that the inflammatory pathways activated through NF-*κ*B signaling play an important role in the development and progression of cancers such as breast, endometrial, and ovarian cancers [106–109].

Insulin resistance and hyperinsulinemia in T2DM promote subclinical or low-grade chronic inflammation that aggravates insulin resistance [110, 111]. Ovarian steroid hormones, particularly estrogen, can activate NF-*κ*B signaling, which induces the gene expression of inflammatory mediators such as IL-1, TNF*α*, and metalloproteinases (MMPs), thereby facilitating inflammatory processes [108, 112, 113]. As noted above, females with T2DM-increased bioavailable ovarian steroid hormones show more enhanced inflammatory effects. Therefore, a chronic inflammatory state in these patients may be the main mechanism associated with cancer development and progression.

## 3. The Link between Diabetes Mellitus and Cancer in Women

The risk of cancers in the female reproductive organs is increased in T2DM. Both breast and endometrial cancer risks are increased in diabetic females. Several biological mechanisms may be involved, mostly regarding ovarian steroid hormone abnormalities.

### 3.1. Breast Cancer

T2DM and breast cancer are both serious life-threatening diseases globally. Breast cancer, the most common cancer and second leading cause of cancer-related death in women, shares some risk factors with diabetes, such as age and obesity [114, 115]. Female diabetic patients were more likely to have increased risk of and mortality from breast cancer [3, 116–118]. A meta-analysis of 20 studies (five case-control and 15 cohort studies between 1966 and February 2007) indicated that females diabetics had an increased risk of breast cancer with a relative risk (RR) of 1.20 [95% confidence interval (CI): 1.12–1.28] [11]. A recently conducted meta-analysis of 12 studies (five case-control and seven cohort studies between 2000 and March 2010) showed a similar result in which women with diabetes have a significantly increased risk of breast cancer with a summary RR of 1.72 (95% CI: 1.47–2.00). Additionally, among postmenopausal or postmenopausal-age females, a strong relationship was demonstrated between diabetes and breast cancer with a summary RR of 1.25 (95% CI: 1.20–1.29) [119]. The Cancer Prevention Study II demonstrated that the incidence of diabetes in females was associated significantly with a 16% increased mortality from breast cancer with an age-adjusted RR of 1.24 (95% CI: 1.11–1.39) and multivariable-adjusted RR of 1.16 (95% CI: 1.03–1.29) [3]. These findings are similar to those of two retrospective cohort studies in the United Kingdom (UK) and Taiwan. Data in the UK study showed a reduced survival time for women with both diabetes and breast cancer (nondiabetes versus diabetes, 14.3 versus 10.4 years, resp.) [117]. In the Taiwan study, breast cancer patients with diabetes had a significantly increased mortality with a hazard ratio (HR) of 1.57 (95% CI: 1.15–2.15) [118].

In breast cancer, insulin and IGFs play important roles as mitogens. Breast cancer tissues showed increased levels of the “A” isoform of the IR (IR-A) activated by insulin and IGF [120]. PI3K and/or Ras/MAPK pathways induced through IR-A activation resulted in mitosis of breast cancer cells in vitro [53]. In vivo studies using nonobese, insulin-resistant, and hyperinsulinemic transgenic MKR mouse models showed that hyperinsulinemia results in mammary ductal hyperplasia and IR-expressing carcinoma [121]. Women with diabetes displayed hormonal changes resulting from increased production of estrogen and androgen with decreased liver production of SHBG [122]. These hormonal changes were also strongly associated with breast cancer risk in postmenopausal females [69]. Increased bioavailable estrogen stimulated the proliferation of ER-positive and/or estrogen-dependent breast cancer [123]. Hyperinsulinemia in T2DM induced the expression and increased the binding capacity of ER [124, 125]. The activation of ER can also enhance insulin mitogenicity by promoting IRS-1 function and activating PI3K and Ras/MAPK signaling [126]. The inflammatory mediators, TNF*α* and IL-6, which are associated with insulin resistance in T2DM, enhanced estrogen production in both normal and breast cancer cells and could be expected to result in the development and proliferation of breast cancer cells [127].

### 3.2. Endometrial Cancer

Endometrial cancer is the most common gynecological cancer and is closely associated with endometrial hyperplasia, unopposed estrogen exposure, and genetic alterations [128, 129]. This cancer has been associated strongly with T2DM in most epidemiological studies [13, 15, 117, 118, 130–132]. Many studies have suggested that T2DM and endometrial cancer share characteristics regarding the major modifiable determinates, such as low physical activity and obesity [133–135]. A meta-analysis of 16 studies (13 case-control and three cohort studies between 1956 and June 2005) indicated that T2DM had a significantly increased risk and comorbidity with endometrial cancer with a summary RR of 2.10 (95% CI: 1.75–2.53). The risk was particularly strong among studies with age-adjusted estimates (RR 2.74; 95% CI: 1.87–4.00) [13]. Also, a recent population-based and retrospective cohort study demonstrated that endometrial cancer and diabetes were strongly associated, with an HR of 1.81 (95% CI: 1.37–2.41), and had an increased relationship, with an age-adjusted HR of 1.85 (95% CI: 1.36–2.50) [15]. Although some studies have shown that endometrial cancer and diabetes had no significant statistical association with mortality [117], most studies have indicated that diabetes is associated with an increased risk of death from endometrial cancer; for example, a prospective study reported a multivariable-adjusted RR of 1.33 (95% CI: 1.08–1.65) and an age-adjusted RR of 1.72 (95% CI: 1.40–2.12) [3].

Similar to breast cancer cells, in vitro studies have shown that endometrial cancer cell lines increased proliferation by activation of insulin, IGF-1, and ovarian steroid hormone signaling pathways, such as estrogen and androgen signaling pathways [54]. Although endometrial cancer has no direct correlation with insulin or IGF levels, additional factors such as ovarian steroid hormones and/or inflammatory cytokines may make it difficult to confirm a single effect of insulin or IGF activation through insulin or IGF serum levels. Estrogen can activate IGF1R on endometrial cancer cells, thereby enhancing cellular proliferation through PI3K signaling, a link to IGF1R activation [136]. The androgen receptor (AR) activated by the binding of androgen could enhance the proliferation of endometrial cancer cells by the Notch signaling pathway [93]. C-reactive protein (CRP), which is an inflammatory biomarker induced by IL-6, was increased by insulin resistance and was associated with an increased risk of endometrial cancer in postmenopausal women [137]. Therefore, endometrial cancer may be associated with chronic inflammation in T2DM.

### 3.3. Ovarian Cancer

Although ovarian cancer is the ninth most common cancer and represents the fifth leading cause of death in women worldwide [138], studies concerning the relationship between ovarian cancer and T2DM are limited. One reason could be due to well-known and very important factors such as familial history, genetic mutations, menstrual cycles, and usage of oral contraceptives [139, 140]. However, some incidences in which the ovary displays insulin sensitivity and steroidogenesis induced by insulin and IGFs suggest that T2DM may be an important risk factor for ovarian cancer [141, 142]. Although small-scale epidemiological studies have demonstrated inconsistent results regarding the relationship between ovarian cancer and T2DM, a recent meta-analysis of 19 studies (six case-case control, one nested case-control, and 12 cohort studies between 1976 and 2007) indicated that women with diabetes had an increased risk of ovarian cancer with a summary RR of 1.17 (95% CI: 1.02–1.33) [3, 5, 117, 118, 143–146]. Many epidemiological studies have shown that ovarian cancer is associated with increased serum androgen levels and decreased serum progesterone levels rather than altered serum estrogen levels [79, 147]. These hormonal changes appear in diabetes and may be one reason for the increased risk of ovarian cancer in T2DM.

Although there is no experimental evidence for the positive association between insulin and ovarian cancer, some studies have shown that the increased serum levels of IGF-1, IGF-1R, and IGFBP-2 were associated positively in patients with ovarian cancer [55]. One study demonstrated that IGF-1 in human ovarian OVCAR-3 cells enhanced the expression of KCl cotransport (KCC) and was associated with proliferation and invasiveness of ovarian cancer cells [148]. Other studies have also shown that IGF-1 and IGFBP-2 in human ovarian cancer cell lines resulted in the induction of proliferation and invasion through phosphorylation of AKT and ERK1/2 [149, 150]. The significance of inflammation in ovarian carcinogenesis stems from the relationship between increased ovulation and ovarian cancer risk [151]. The role of androgen in stimulating the proliferation of ovarian cancer cells may also be associated with increased IL-6 and decreased transforming growth factor beta (TGF*β*), which were included in the proinflammatory network [92, 152].

## 4. The Role of Diabetes Medications in Cancer Development in Women

The potential effects of antidiabetic medications on cancer have sparked recent discussion and concern among the epidemiological and experimental studies related to the potential underlying mechanisms. In this section, we discuss the relationship between cancer risk and antidiabetic medication, including insulin and insulin analogs, metformin, and thiazolidinediones.

### 4.1. Insulin/Insulin Analogues

As noted above, because excessive insulin and IGF-1 signaling by hyperinsulinemia may be one of the most important causes of the development and proliferation of cancer, exogenous insulin is a suspected powerful carcinogenetic factor in diabetes patients. Increased circulating insulin levels over endogenous insulin secretion occur frequently with subcutaneous insulin injection, thereby making possible the association between insulin therapy and cancer [21, 153, 154]. Nevertheless, all patients with type 1 DM (T1DM) and approximately 40~80% of patients with T2DM are considered for insulin therapy to maintain proper glycemic control [155].

A significant number of epidemiological studies have suggested that insulin use and daily doses, particularly of the long-acting insulin analog glargine, may be responsible for the association with and strong increase in the risk of cancer [156–160]. A recent meta-analysis of 15 studies (five case-control and 10 cohort studies) demonstrated that insulin treatment was associated significantly with an increased risk of overall cancer with a summary RR of 1.39 (95% CI: 1.14–1.70), particularly in case-control studies that evaluated T1DM, with a higher summary RR of 1.83 (95% CI; 0.99–3.38) [161]. Some studies showed a strong relationship between insulin glargine and breast cancer, particularly in T2DM patients treated with insulin for more than 5 years [162, 163]. Only a few studies with large-scale patient databases exist, such as the ORIGIN trial which enrolled 12,537 patients and followed them for 6.2 years (interquartile range, 5.8–6.7 years) and showed no statistically significant association between cancer risk and insulin glargine use; however, these results may be due to very well-controlled glucose levels, as well as the inclusion of prediabetic patients [9, 164]. Therefore, optional selection between insulin treatment and proper glucose control may be needed for patients with diabetes, particularly T2DM.

### 4.2. Metformin

Metformin is an oral antidiabetic drug classified as an insulin sensitizer and is the most widely used drug, prescribed as the initial or in combination therapy, for T2DM [165]. Metformin reduces serum glucose and insulin levels in diabetic patients via improved insulin sensitivity, which reduces glucose production in the liver and increases glucose uptake in the muscles [166, 167]. Another feature of metformin that attracts special attention is its anticancer effects supported by evidence from epidemiologic, in vitro, and in vivo model studies [3, 168–175].

In in vitro studies, metformin inhibits complex I of the mitochondrial respiratory chain, resulting in ATP/AMP imbalance, thereby activating AMP-activated protein kinase (AMPK) [176]. Activated AMPK inhibits mTOR signaling and interferes with the roles of cyclin D1 and p53. The latter two proteins not only alter glucose metabolism but also reduce cell proliferation through cell cycle interference [177–181].

The anticancer effects of metformin have been shown to reduce spontaneous mammary tumor development in rodent animal models [182, 183]. However, some studies using mouse models found that metformin induced insulin resistance and hyperinsulinemia, which suggested that the anticancer effect of metformin may be mediated by reduced serum insulin levels [184]. In other words, the insulin-lowering effect of metformin was associated with its anticancer effect, thereby having less of an impact on cancer in patients with normal or lower insulin levels.

Most epidemiological studies have suggested that metformin used for T2DM reduces the risk, progression, and mortality of overall cancer [169, 171, 185–189]. Although those studies were limited in their assessment of a detailed relationship between metformin and specific cancer types, a recent meta-analysis of 28 studies showed that metformin has a significant inverse association with cancer mortality, including endometrial and ovarian cancers [187].

### 4.3. Thiazolidinediones

Thiazolidinediones (TZDs) are insulin-sensitizing antidiabetic drugs belonging to the peroxisome proliferator-activated receptor (PPAR) agonist class that induce the transcription of genes associated with glucose and lipid metabolism through activation of PPAR*γ*, a nuclear receptor, to reduce insulin resistance [190]. The effects of TZDs in reducing insulin resistance may be expected to result in anticancer activities similar to those of metformin. Some studies have indicated antiangiogenic and anti-inflammatory effects of TZDs, as well as anticancer effects, such as the inhibition of proliferation and induction of apoptosis and differentiation [191]. However, unlike metformin, the effects of TZDs are inconsistent between in vitro and in vivo studies and depend on parameters such as the animal model (rodent versus nonrodent and nonhuman primate versus human) and cancer type [192–197]. Some studies in rodents have suggested that TZDs can even potentiate tumorigenesis as multispecies and multisex carcinogens [192, 198–200].

Epidemiological studies evaluating the relationship between TZDs and cancer risk are limited and have shown inconsistent results [201–205]. One meta-analysis of randomized clinical trials concerning rosiglitazone and cancer risk indicated that rosiglitazone did not alter the risk of cancer, including breast and female genital tract cancers [206]. A recent meta-analysis indicated that pioglitazone, but not rosiglitazone, was associated significantly with a decreased risk of breast cancer (Mantel-Haenszel odds ratio (MH-OR): 0.28 [0.09–0.93]; *P* = 0.038), but neither pioglitazone nor rosiglitazone altered the risk of uterine cancer (MH-OR: 0.77 [0.34–1.73]; *P* = 0.52) [207].

## 5. Conclusion

The incidence and prevalence of gynecologic cancers are increased in patients with T2DM. Similar to other cancers, gynecologic cancers have several common mechanisms with T2DM, including increased insulin and IGF signaling and chronic inflammation. Unlike other cancers, dysregulation of ovarian steroid hormones is another commonly associated mechanism between T2DM and gynecologic cancer. Insulin resistance could induce or aggravate ovarian steroid hormone dysregulation and chronic inflammation in diabetic women. Most epidemiological studies have suggested that cancer in diabetic women can be modulated by insulin sensitizers such as metformin and TZDs. The management of insulin resistance is a main factor in controlling blood glucose and preventing cancer in female diabetic patients.

Thus, clinicians should recommend life-style changes such as weight-loss diets and exercise to overcome insulin resistance in women with T2DM. Additionally, clinicians should attend to and perform screening tests for gynecologic cancers according to currently established routines until screening protocols are developed for each specific gynecologic cancer in women with T2DM.
